# AGE INVARIANCE IN THE FACTOR STRUCTURE OF ADVERSE CHILDHOOD EXPERIENCES ACROSS THE LIFESPAN

**DOI:** 10.1093/geroni/igad104.3018

**Published:** 2023-12-21

**Authors:** Amber Rusch, Julie Hicks Patrick

**Affiliations:** West Virginia University, Morgantown, West Virginia, United States; West Virginia University, Morgantown, West Virginia, United States

## Abstract

Adverse Childhood Experiences (ACEs) are associated with negative health and well-being across the adult life span. However, it is unclear whether measures of ACEs are equally predictive for adults of differing ages. Using responses from 125,212 adults on the 11-items in the ACEs Module from the 2020 Behavioral Risk Factor Surveillance System (BRFSS), we conducted both an exploratory factor analysis (EFA) and confirmatory factor analysis (CFA). Our EFA identified three interpretable factors, including a 3-item sexual abuse factor (α = .80), a 3-item physical/emotional abuse factor (α = .69) and a 5-item household dysfunction factor (α = .64). Results of our CFA supported the 3-factor solution, X2 (DF = 11) = 14.289, p < .001. A series of 1-way ANOVAS examined age differences in childhood adversity, where middle aged adults reported experiencing more sexual abuse relative to younger and older adults, and younger adults reported experiencing more physical/emotional abuse and household dysfunction compared to middle-aged who reported more than older adults. Additional analyses examined the influence of adverse childhood experiences on physical and mental health outcomes, including depression. Our analyses highlight the persistent effect of ACEs across the lifespan and identify a need for intervention resources for younger and middle-aged adults in order to mitigate effects of ACEs at late life.

